# Exploring the effects of student leadership on higher vocational students’ social and emotional competence: an empirical study from China

**DOI:** 10.3389/fpsyg.2026.1931236

**Published:** 2026-09-23

**Authors:** Rong Xiang, Min Deng

**Affiliations:** Normal School of Vocational Techniques, Hubei University of Technology, Wuhan, Hubei, China

**Keywords:** Chinese higher vocational students, learning engagement, peer interaction, social and emotional competence, student leadership

## Abstract

**Objective:**

This study examined the relationship between student leadership and social and emotional competence among higher vocational students, and explored the mediating roles of teacher-student interaction, peer interaction, and learning engagement.

**Methods:**

Survey data were collected from 1,895 higher vocational students in Hubei Province, China. Propensity score matching was used to reduce observable selection bias between students with and without leadership roles, and bootstrap mediation analyses were used to test the indirect pathways.

**Results:**

Students holding leadership roles reported higher social and emotional competence than matched students without leadership roles. Teacher-student interaction, peer interaction, and learning engagement all showed significant mediating roles, with learning engagement showing the largest indirect effect.

**Conclusion:**

Student leadership is positively associated with higher vocational students’ social and emotional competence. The findings suggest that leadership roles may function not only as symbolic status, but also as role practice involving communication, coordination, and engagement.

## 1 Introduction

Social and emotional competence has become an important way to understand students’ academic adjustment, interpersonal adaptation, and long-term development. Its cultivation is associated with greater adaptability, stronger social support, improved academic development, and better long-term outcomes (Elias and Haynes, 2008; Heckman and Kautz, 2012; Jones and Kahn, 2017; Durlak et al., 2011; Taylor et al., 2017; OECD, 2021). Within the OECD framework, social and emotional competence includes task performance, emotional regulation, collaboration, open-mindedness, and engagement with others, reflecting a multidimensional set of capacities that help individuals manage goals, regulate emotions, build relationships, and adapt to changing environments (OECD, 2015, 2021). For higher vocational students, this competence is especially practical: they must adapt not only to classroom learning, but also to practical training, peer collaboration, teacher communication, and future workplace transitions. In China, projections suggest that by 2030, demand for physical and manual skills will decline, whereas demand for social and emotional skills will increase substantially (Woetzel et al., 2021). This shift makes it necessary for higher vocational education to look beyond narrow technical training and to attend more closely to the psychological, relational, and motivational conditions that support the development of students’ social and emotional competence.

Student leadership offers one such experience. As a common form of role participation in Chinese educational institutions, student leadership links institutions, teachers, and students, and has been increasingly recognized as a meaningful context for students’ holistic development (Qian, 2025). In Chinese higher vocational colleges, students who serve in leadership roles, such as class monitors, league secretaries, study committee members, or other student cadres, usually participate in class management, communicate with teachers, coordinate peer activities, and support the implementation of school and class affairs. Unlike many Western student leadership contexts, student leaders in China are often selected through procedures that value not only academic performance, but also responsibility, diligence, interpersonal competence, and organizational capacity (Du, 2016). These roles are therefore more than administrative assignments. They may become role-based developmental contexts in which students receive recognition and expectations from teachers and peers while repeatedly practicing communication, coordination, responsibility-taking, and participation.

Existing studies have suggested that students with leadership experience tend to show stronger organizational, interpersonal, and responsibility-related qualities (Cui et al., 2022; Qian, 2025; Seemiller, 2018). Leadership participation may also provide students with greater autonomy and decision-making opportunities, allowing them to cultivate competencies through authentic learning experiences (Seemiller, 2018). Yet it remains unclear whether these observed advantages remain associated with student leadership after accounting for pre-existing differences among students who are more likely to be selected as leaders. Prior research has also tended to focus on isolated outcomes such as communication skills, academic performance, career prospects, or specific leadership competencies, while giving less attention to social and emotional competence as an integrated and multidimensional developmental outcome (Kelley, 2008; Seemiller, 2018). This limitation is particularly salient in higher vocational education, where students’ development is closely connected with technical training, school-to-work transition, and practical participation in institutional and peer communities.

This study conceptualizes student leadership as a role-based developmental context and considers its developmental relevance from two complementary perspectives: the symbolic meaning attached to formal leadership status and the practical experiences generated through role participation. Self-Determination Theory further provides a theoretical basis for examining how the relational and motivational experiences associated with leadership participation may be relevant to social and emotional competence (Deci and Ryan, 2000; Reeve, 2012; Ryan and Deci, 2017, 2020).

Using survey data from higher vocational students in Hubei Province, China, the study first examines the association between formal student leadership status and social and emotional competence after reducing observable selection bias, and then examines three potential relational and motivational pathways. Together, these analyses connect formal leadership status with the experiences accompanying leadership participation.

## 2 Literature review

### 2.1 Student leadership as symbolic status and role practice

In China, student leaders are typically selected through democratic, class-level procedures to undertake organizational responsibilities and pursue collective goals (Ke and Li, 2020). Positioned between institutional administration and student self-governance, they often act as intermediaries who support class management, facilitate communication between teachers and students, and represent peer groups in school affairs (Du, 2016). In this sense, student leadership is not simply an administrative arrangement. It is a role-based experience embedded in the everyday organization of school life.

Existing studies have documented several benefits associated with student leadership. Some of these benefits are linked to the symbolic status attached to leadership roles. Holding a leadership position often gives students greater visibility, recognition, and expectations from teachers and peers, which may increase their access to learning resources and developmental opportunities, such as greater classroom participation, academic recognition, and institutional support (Ke and Li, 2020; Skalicky et al., 2020). From this perspective, student leadership can operate as a form of symbolic capital, through which students gain status-based advantages that may strengthen their sense of responsibility, self-efficacy, and role identity (Bourdieu, 1986; Cui et al., 2022).

Other benefits are more closely related to role practice. Student leaders are usually required to organize class affairs, coordinate collective activities, communicate with teachers, and respond to classmates’ needs. These repeated practices give students concrete opportunities to develop communication, coordination, perspective-taking, and self-regulation. Prior research has shown that leadership participation is associated with stronger organizational coordination, collaborative communication, academic performance, and psychological well-being (Cui et al., 2022; Deng et al., 2020; Qian, 2025). The developmental significance of student leadership may therefore lie not only in the status it confers, but also in the relational and practical experiences it creates.

Previous research, however, has often treated student leadership as a single status variable, without clearly distinguishing the symbolic status of holding a leadership position from the role-practice opportunities generated by leadership participation. This distinction matters because symbolic status may shape students’ self-understanding through recognition and expectations, whereas role practice may expose students to repeated interactions, responsibilities, and learning-related engagement. This distinction provides the conceptual basis for examining the association attached to formal leadership status alongside the relational and motivational processes associated with leadership participation.

### 2.2 Student leadership and social and emotional competence of higher vocational students

Student leadership may be associated with social and emotional competence because it places students in a visible and responsible role within peer and institutional communities. In higher vocational colleges, leadership participation is closely connected with communication, coordination, responsibility-taking, and participation in collective affairs. These experiences are directly relevant to interpersonal competence, self-regulation, cooperation, and socially adaptive behaviors, all of which are central components of social and emotional competence.

Empirical evidence regarding the relationship between student leadership and student development remains mixed. Some studies have reported that student leaders tend to demonstrate stronger communication, organizational, and self-regulation competence than their non-leader peers (Mumford et al., 2000; Zhang and Gan, 2020). Cui et al. (2022) further observed that leadership participation is associated with task performance and interpersonal competence through active engagement and responsibility-taking. Other studies have found no significant differences in perseverance, stress resilience, or emotional stability between leaders and non-leaders (Li and Wang, 2019). There are also concerns that leadership roles may bring emotional overload, role pressure, and anxiety when students face excessive workload or high expectations (Zhou, 2014; Zhu, 2014).

These inconsistent findings make the association between student leadership and social and emotional competence worth examining more carefully. First, much prior research has focused on isolated traits, such as communication, perseverance, or stress tolerance, rather than treating social and emotional competence as a multidimensional developmental outcome. This narrow approach may overlook how leadership participation is differently associated with task performance, emotional regulation, collaboration, open-mindedness, and engagement with others. Methodologically, because student leadership is not randomly assigned, the present study uses propensity score matching to reduce observable background differences between students with and without leadership roles before examining their differences in social and emotional competence.

### 2.3 Role-practice pathways from a self-determination theory perspective

Self-Determination Theory (SDT) provides a useful framework for understanding why the relational and motivational processes associated with the role practice of student leadership may be linked to social and emotional competence. According to SDT, students’ motivation and development are supported when their basic psychological needs for competence, relatedness, and autonomy are fulfilled (Ryan and Deci, 2000; Ryan and Deci, 2020). Competence refers to students’ perceived effectiveness in accomplishing meaningful tasks; relatedness reflects their sense of connection and support in social relationships; and autonomy refers to acting with volition and self-endorsement rather than external pressure (Niemiec and Ryan, 2009; Ryan and Deci, 2020). SDT has often been applied to teacher support, classroom climate, and learning motivation. Student leadership offers a useful extension of this perspective because it places students in active roles within school life rather than treating them only as recipients of motivational support.

In higher vocational colleges, the role-practice dimension of student leadership may create relational and motivational experiences that are aligned with these psychological needs. Teacher-student interaction may provide competence-supportive experiences: student leaders often communicate with teachers about class affairs, academic tasks, and collective activities, and through these interactions they may receive feedback, recognition, and guidance that strengthen their sense of responsibility and perceived competence (Du, 2016; Collie and Martin, 2024). Peer interaction may provide relatedness-oriented experiences. Leadership roles require students to coordinate peer activities, mediate group affairs, and respond to classmates’ needs, creating opportunities for cooperation, belonging, empathy, and mutual support (Collie and Martin, 2024; Ryan and Deci, 2020). Learning engagement may represent a further motivational pathway because leadership roles often involve responsibility-taking, self-regulation, and participation in collective decision-making, which may encourage students to invest more effort and attention in learning-related activities (Christenson et al., 2012; Deng et al., 2020).

Taken together, Self-Determination Theory provides a theoretical lens for understanding how the relational and motivational experiences associated with student leadership may be linked to social and emotional competence.

### 2.4 The current study and hypotheses

Building on this discussion, the present study examines the association between student leadership and social and emotional competence among higher vocational students in Hubei Province, China. Empirically, the study proceeds in two steps. First, it examines the association between formal student leadership status and social and emotional competence, with the symbolic-status perspective informing the interpretation of the developmental meaning attached to holding a recognized leadership position. Second, it examines teacher-student interaction, peer interaction, and learning engagement as potential mediating processes interpreted from a role-practice perspective. Self-Determination Theory provides theoretical guidance for understanding these relational and motivational pathways. Given that student leadership selection is not random, propensity score matching is used to reduce observable selection bias before examining the association between leadership status and social and emotional competence.

Accordingly, this study proposes the following hypotheses:

Hypothesis 1: Student leadership is positively associated with social and emotional competence among higher vocational students after accounting for observable background characteristics.

Hypothesis 2a: Teacher-student interaction mediates the association between student leadership and social and emotional competence.

Hypothesis 2b: Peer interaction mediates the association between student leadership and social and emotional competence.

Hypothesis 2c: Learning engagement mediates the association between student leadership and social and emotional competence.

Drawing on this literature and theoretical perspectives, a conceptual framework is proposed, as illustrated in Figure 1.

**Figure 1:**
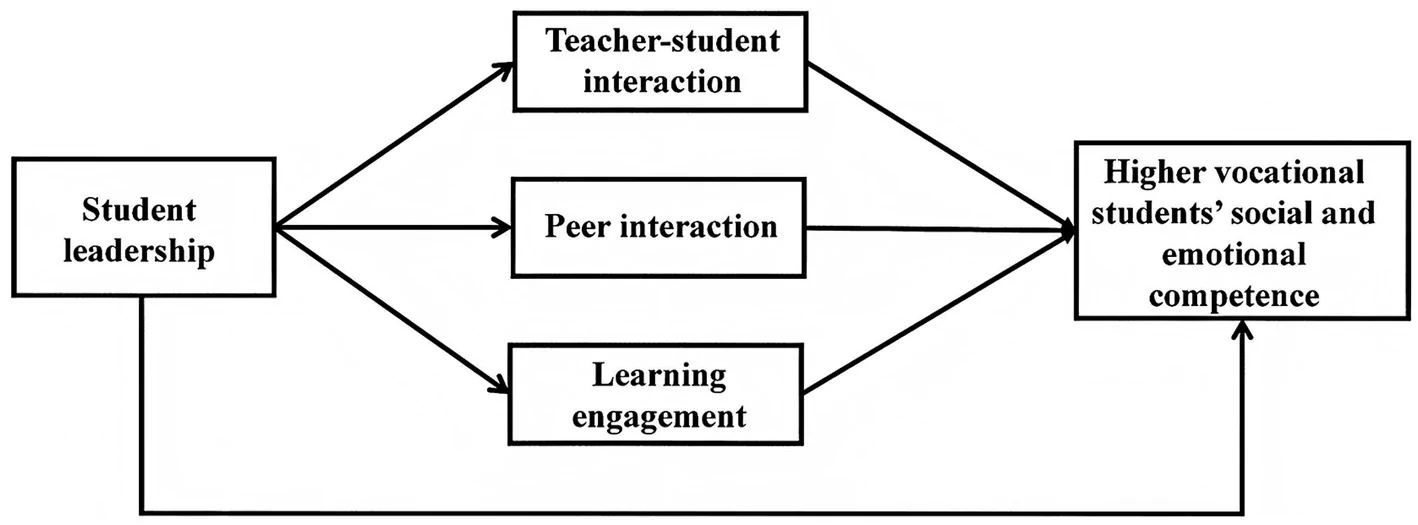
Modeling framework.

## 3 Methods

### 3.1 Procedure

In September 2023, a survey was conducted to assess the social and emotional competence of students in higher vocational institutions in Hubei Province, China. Hubei Province is a pivotal region for education, technology, and manufacturing, whose geographical advantages, historical resources, and sustained development have provided a strong foundation for vocational education. By 2023, the province hosted 63 higher vocational institutions, 120 technical schools, and 252 secondary vocational schools (Hubei Provincial Statistics Bureau, 2024), forming one of the largest vocational education systems in China. With a relatively large and diverse higher vocational education system comprising institutions with different governance types and resource levels, Hubei provides a useful regional context for examining student leadership experiences within Chinese higher vocational education. These institutions play a vital role in promoting socioeconomic development by training a large number of skilled professionals.

The study employed a stratified random sampling design. To ensure that the sample accurately represented the complexity and resource distribution of higher vocational institutions in Hubei Province, all institutions were initially classified according to two primary stratification criteria. The first criterion distinguished public from private institutions, while the second differentiated institutions based on their prestige and resource allocation, categorizing them as either “Double High-level Plan” institutions or regular institutions. Proportional stratified random sampling was subsequently employed to select institutions from each of the four resulting categories. Within each selected institution, students were sampled using a two-stage random procedure. In the first stage, majors were grouped into natural sciences/engineering and humanities/social sciences. In the second stage, administrative classes within these categories were randomly selected.

Data were collected between August and September 2023 using “Questionnaire Star,” a widely used online survey platform in Chinese higher education institutions. The anonymous questionnaire included items on students’ demographics, family background, and campus life. A total of 2070 students from 24 higher vocational institutions participated. To ensure data quality and integrity, strict control measures were implemented on the “Questionnaire Star” platform. First, a duplication control mechanism was established to restrict each device or IP address to a single submission, preventing multiple responses from the same participant. Second, a time-based screening procedure was applied, automatically excluding questionnaires completed in under 2 min, a threshold representing less than one-third of the median completion time (6.3 min), to eliminate inattentive responses. Finally, pattern screening was conducted, and all questionnaires exhibiting identical responses across all Likert-scale items were manually removed. Following these quality control procedures, a total of 1895 responses were deemed valid, corresponding to an effective response rate of 91.5%. The final sample consisted of 1895 students, including 999 male (52.7%) and 896 female (47.2%). Regarding household registration status, 1,280 students (67.5%) had rural status, and 615 (32.5%) had non-rural status.

The study used an anonymous educational questionnaire survey. Before completing the questionnaire, participants were informed of the purpose of the study, the intended use of the data, the anonymous nature of the survey, and their right to withdraw at any time. No personally identifiable information was collected. Oral informed consent was obtained from all participants before their participation. The study protocol and the use of anonymized survey data were reviewed and approved by the Scientific Research Ethics and Technology Safety Committee of Hubei University of Technology, which confirmed that the study complied with institutional ethical requirements for low-risk educational survey research.

### 3.2 Instruments

#### 3.2.1 Dependent variables

The dependent variable, social and emotional competence, was measured using a 46-item scale based on the OECD framework for social and emotional competence, which integrates the Big Five Personality model and ecosystem theory. The scale assessed five dimensions, each corresponding to a major personality domain: task performance (9 items; e.g., “I keep working on a task until it is finished”; α = 0.82), emotional regulation (10 items; e.g., “I stay calm even in tense situations”; α = 0.80), open-mindedness (9 items; e.g., “I am curious about many different things”; α = 0.92), collaboration (9 items; e.g., “I cooperate very well with others”; α = 0.89), and engaging with others (9 items; e.g., “I usually feel full of energy”; α = 0.83). Items were rated on a five-point Likert scale (1 = strongly disagree to 5 = strongly agree). The overall scale demonstrated excellent internal consistency (α = 0.94) and good factorability (KMO = 0.981; *p* < 0.001). Confirmatory factor analysis further provided partial support for the hypothesized five-factor structure, χ^2^ = 5004.61, CFI = 0.898, TLI = 0.885, RMSEA = 0.049, and SRMR = 0.051.

#### 3.2.2 Independent variables

The independent variable was formal student leadership status, operationalized as whether a student currently held a student leadership position. This variable was measured with a single binary item: “Are you currently serving as a student leader in the current academic year?” Responses were coded as 1 (Yes) and 0 (No). For the PSM analysis, students with the current leadership status comprised the treatment group, while those without formed the control group. Overall, 56.6% of participants reported holding a student leadership role.

#### 3.2.3 Mediating variables

To examine the potential mediating processes associated with the role practice of student leadership, this study included three school-based relational and motivational variables: teacher-student interaction, peer interaction, and learning engagement. These variables were selected as observable relational and motivational experiences theoretically relevant to Self-Determination Theory. They were not intended as direct measures of competence, relatedness, or autonomy need satisfaction; rather, SDT was used to guide their theoretical interpretation. The items were adapted from large-scale national surveys and prior studies on student development (Tang and Jiang, 2024).

Teacher-student interaction was operationalized as the frequency of counselor–student communication. Students were asked, “How often does your counselor have talks or communicate with you?” Responses were rated on a 5-point Likert scale ranging from 1 (never) to 5 (always). Higher scores indicated more frequent teacher-student interaction. In Chinese higher vocational colleges, counselor-student communication represents an institutionally salient form of teacher-student interaction.

Peer interaction was operationalized as students’ participation in structured peer activities. Students responded to the item: “How often do you participate in club activities with your classmates?” Responses were rated on a 4-point scale ranging from 1 (never) to 4 (weekly). Club participation was used as an observable indicator of structured peer interaction because it captures repeated participation in collective peer activities.

Following Green et al.’s (2012) framework, learning engagement was represented by research participation and self-directed study time as behavioral indicators, and by study satisfaction and commitment as emotional indicators. Because the four indicators were measured on different response scales, principal-component factor analysis was conducted on the correlation matrix so that the indicators entered the extraction procedure on a common standardized metric. The index was calculated as follows:

$$\text{Learning engagement}=\frac{\left(\beta_{1}\mathrm{act}+\beta_{2}\text{time}+\beta_{3}\mathrm{sat}+\beta_{4}\mathrm{pre}\right)}{\gamma }$$

Where act denotes participation in research activities, time denotes self-directed study time, sat denotes satisfaction with studies, and pre denotes commitment to studies. $\beta_{1}$ to $\beta_{4}$ represent the factor loadings of the four indicators, and γ denotes the eigenvalue of the retained principal factor. The numerator therefore represents the factor-loading-weighted combination of the four engagement indicators, while division by γ was used to normalize the weighted composite by the eigenvalue of the retained factor. Higher values indicate stronger learning engagement.

#### 3.2.4 Covariates

To reduce observable selection bias, this study controlled for a set of pre-treatment covariates that may be associated with both the likelihood of holding a student leadership role and students’ social and emotional competence (Du, 2016; Tang, 2017; Li and Wang, 2019; Ke and Li, 2020; Kim et al., 2020; Cui et al., 2022; Wang et al., 2025). These variables were included in the propensity score model because they represent individual, family, and institutional background characteristics that theoretically precede leadership participation.

Individual-level covariates included gender (male = 1, female = 0), year level, household registration status (hukou; rural = 1, non-rural = 0), left-behind experience, and only-child status. Family-level covariates included parental education, parental occupation, household economic status, and household cultural capital. Household cultural capital was measured by the number of books at home. School-level covariates included institutional type, coded as 1 for “Double High Plan” institutions and 0 for other institutions. Where available, student-faculty ratio was also included to account for institutional resource differences.

### 3.3 Statistical analyses

First, we assessed the baseline comparability of the two groups (students with and without leadership experience), to ensure the initial comparability of the groups.

Second, PSM was employed to estimate the Average Treatment Effect on the Treated (ATT), which in this study is interpreted as the matched difference in social and emotional competence associated with holding a student leadership role. This method matched each student with a leadership role to one or more peers without such roles based on similar observable characteristics, thereby improving the comparability of the two groups (Rosenbaum and Rubin, 1985). Because the data were cross-sectional and unobserved selection could not be fully ruled out, the ATT estimates were treated as adjusted associations rather than definitive causal effects. The ATT was estimated using the following equation:

$$\mathrm{ATT}=E\{E[L_{1i}-L_{\mathrm{0i}}|D_{i}=1,p(X_{i})]\}$$

Let $L_{1i}$ and $L_{0i}$ represent the potential social and emotional competence scores for students i when serving and not serving as a student leader, respectively. Let $D_{i}$ be a binary treatment variable indicating leadership position, where $D_{i}=1$ if student i has student leadership position and $D_{i}=0$ otherwise. The propensity score for student i , denoted by $p(X_{i})$ , is defined as the conditional probability that student i has leadership position given a set of observed covariates X . We primarily employed the k-nearest neighbor (KNN) matching with k = 4 and matching with replacement. The caliper was set at 0.01 standard deviations of the propensity score to ensure tight matches and minimize bias. Radius matching and non-parametric kernel matching were employed as robustness checks.

Third, to examine the mediating mechanisms linking leadership status to SEC, we employed a regression-based approach with a bias-corrected bootstrap procedure. To ensure consistency and comparability across models, all continuous mediating and outcome variables were first standardized into z-scores. We used a bias-corrected bootstrap method with 5,000 replications to estimate the 95% confidence intervals for the indirect effects of the three relational mediators. Mediation effects were considered statistically significant if the 95% bias-corrected bootstrap confidence intervals for the indirect effects did not include zero. In addition, Harman’s single-factor test was conducted as a post-hoc diagnostic for potential common method variance arising from the single-source self-report design. The first unrotated factor accounted for 48.31% of the total variance, indicating that no single common factor dominated the variance.

## 4 Results

### 4.1 Descriptive statistics and baseline differences

Before implementing propensity score matching, we examined descriptive differences between students with and without student leadership. As shown in Table 1, the two groups differed in several background characteristics, proposed mediating variables, and outcome measures. Students with student leadership were more likely to be female and to come from families with higher levels of household cultural capital. They also reported more frequent teacher-student interaction, peer interaction, and learning engagement. On the outcome side, students with student leadership showed higher overall social and emotional competence than those without such roles. At the dimensional level, differences were observed in task performance, emotional regulation, collaboration, open-mindedness, and engaging with others. These baseline differences indicate that simple comparisons between the two groups may be affected by observable selection bias, thereby supporting the use of propensity score matching.

**Table 1:** Descriptive results (pre-matching).

Variables	Full sample (*N* = 1895)	Non-student leader (*N* = 822)	Student leader (*N* = 1,073)	Independent samples *t*-test
Student leadership	0.57(0.50)	0	1	-
Social and emotional competence	3.44(0.54)	3.34(0.53)	3.52(0.54)	−0.18***(−7.18)
Task performance	3.5(0.53)	3.41(0.51)	3.57(0.52)	−0.16***(−6.83)
Emotion regulation	3.28(0.55)	3.23(0.53)	3.32(0.56)	−0.09***(−3.7)
Collaboration	3.54(0.66)	3.43(0.66)	3.62(0.65)	−0.19***(−6.24)
Open-mindedness	3.56(0.67)	3.45(0.65)	3.65(0.67)	−0.2***(−6.4)
Engaging with others	3.31(0.59)	3.17(0.58)	3.42(0.58)	−0.25***(−9.13)
Gender	0.53(0.5)	0.59(0.49)	0.48(0.5)	0.11***(4.92)
Household registration	0.68(0.47)	0.69(0.46)	0.66(0.47)	0.03(1.17)
Left-behind experience	0.39(0.49)	0.38(0.49)	0.40(0.49)	−0.01(−0.57)
Grade level	1.96(0.77)	2.03(0.74)	1.90(0.79)	0.13***(3.6)
Parental education	10.31(2.84)	10.27(2.83)	10.35(2.84)	−0.08(−0.61)
Parental occupation	3.78(1.81)	3.73(1.78)	3.82(1.83)	−0.09(−1.1)
Household book collection	3.26(1.28)	3.14(1.25)	3.36(1.30)	−0.22***(−3.71)
Household economic level	1.92(0.62)	1.91(0.59)	1.92(0.63)	−0.02(−0.59)
Learning engagement	2.51(0.52)	2.43(0.51)	2.58(0.52)	−0.15***(−6.14)
Peer interaction	2.5(1.26)	2.25(1.27)	2.70(1.22)	−0.45***(−7.87)
Teacher-student interaction	2.42(0.99)	2.32(1.00)	2.49(0.97)	−0.17***(−3.78)
Pupil-teacher ratio	14.13(3.14)	14.06(3.00)	14.19(3.24)	−0.14(−0.93)
College type	0.75(0.43)	0.74(0.44)	0.76(0.42)	−0.02(−1.17)

Standard deviations are reported in parentheses in the first three columns; Column 4 presents the results of the Standardized mean-difference t-tests, with corresponding t-statistics in parentheses; ^+^p < 0.1, *p < 0.05, **p < 0.01, ***p < 0.001; These notes apply throughout the paper.

### 4.2 Balance diagnostics after propensity score matching

Propensity score matching was used to improve the comparability of students with and without student leadership on observed pre-treatment background characteristics. As shown in Table 2, several covariates, including gender, grade level, and household cultural capital, showed noticeable differences before matching. After matching, these differences became statistically insignificant. The standardized mean differences for all included pre-treatment covariates were reduced to below 5%, suggesting that the matching procedure produced acceptable balance on the observed background characteristics.

**Table 2:** Balance diagnostics for covariates before and after matching.

Variables	Match status	Treatment group	Control group	Bias reduction (%)	Absolute value of reduction (%)	T-Statistic
Gender	Before	0.48	0.59	−22.8	93.70	−4.92***
	After	0.48	0.49	−1.4		−0.33
Household registration	Before	0.66	0.69	−5.4	76.50	−1.17
	After	0.67	0.67	−1.3		−0.29
Left-behind experience	Before	0.4	0.38	2.6	39.30	0.57
	After	0.4	0.39	1.6		0.37
Grade level	Before	1.9	2.03	−16.8	96.00	−3.6***
	After	1.9	1.91	−0.7		−0.15
Parental education	Before	10.35	10.27	2.8	63.70	0.61
	After	10.34	10.31	1		0.24
Parental occupation	Before	3.82	3.73	5.1	61.00	1.1
	After	3.82	3.78	2		0.46
Household book collection	Before	3.36	3.14	17.3	81.70	3.71***
	After	3.35	3.31	3.2		0.73
Household economic level	Before	1.92	1.91	2.8	4.50	0.59
	After	1.92	1.91	2.6		0.61
Pupil-teacher ratio	Before	14.19	14.06	4.3	57.30	0.93
	After	14.18	14.24	−1.8		−0.41
College type	Before	0.76	0.74	5.4	71.70	1.17
	After	0.76	0.76	1.5		0.36

The density plot in Figure 2 provides a visual check of this improved covariate balance. Before matching, the distributions of propensity scores differed between the treatment and control groups. After matching, the two distributions became more similar and showed a larger region of common support. The matched sample therefore provides a more appropriate basis for comparing students with and without student leadership, although balance on observed covariates cannot eliminate bias from unobserved factors.

**Figure 2:**
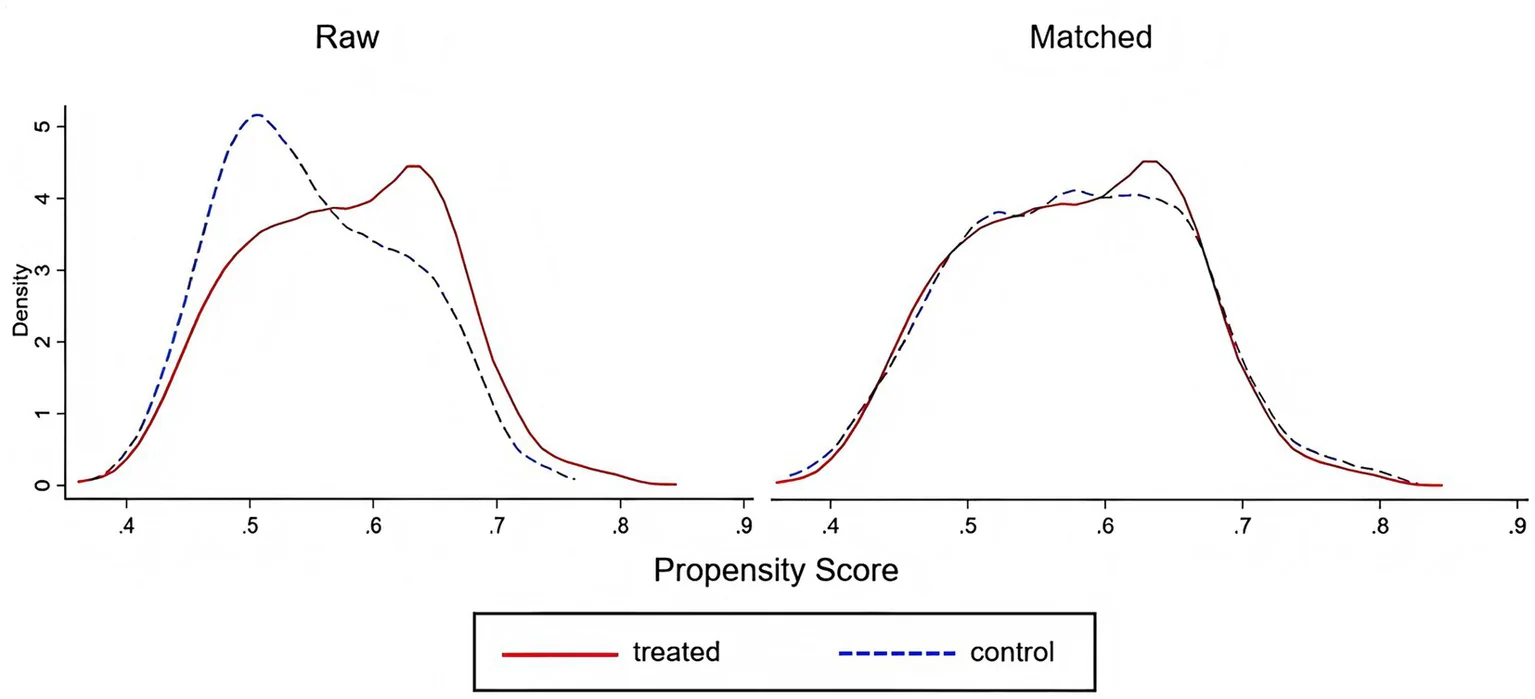
Balancing plot of propensity score matching.

### 4.3 Matched association between student leadership and social and emotional competence

Table 3 reports the estimated differences in social and emotional competence between students with and without student leadership before and after matching. The K-nearest neighbors matching results showed that students with student leadership reported higher overall social and emotional competence than matched peers without such roles. The same pattern appeared across all five dimensions, including task performance, emotional regulation, collaboration, open-mindedness, and engaging with others.

**Table 3:** Average treatment effects on the treated (ATT) for social and emotional competence.

Explained variables	KNN matching					Radius matching		Kernel matching	
	Match Status	Treatment group	Control group	ATT	S.E.	ATT	S.E.	ATT	S.E.
Social and emotional competence	Before	3.517	3.339	0.178***	0.025	0.178***	0.025	0.178***	0.025
	After	3.517	3.318	0.199***	0.033	0.184***	0.026	0.182***	0.025
Task performance	Before	3.573	3.409	0.164***	0.024	0.164***	0.024	0.164***	0.024
	After	3.573	3.389	0.184***	0.032	0.162***	0.025	0.161***	0.025
Emotional regulation	Before	3.324	3.230	0.094***	0.025	0.094***	0.025	0.094***	0.025
	After	3.324	3.212	0.112***	0.033	0.106***	0.026	0.104***	0.026
Collaboration	Before	3.621	3.433	0.188***	0.03	0.188***	0.030	0.188***	0.030
	After	3.623	3.412	0.211***	0.041	0.194***	0.032	0.189***	0.031
Open-mindedness	Before	3.648	3.451	0.196***	0.031	0.196***	0.031	0.196***	0.031
	After	3.647	3.426	0.222***	0.041	0.204***	0.032	0.202***	0.031
Engaging with others	Before	3.417	3.171	0.246***	0.027	0.246***	0.027	0.246***	0.027
	After	3.417	3.152	0.265***	0.036	0.256***	0.028	0.257***	0.028

Columns 1–2 report mean social and emotional competence scores for the treatment and control groups, respectively; ATT represents Average Treatment Effect on the Treated, calculated as the mean difference in social and emotional competence and five dimensions between students with student leadership role and students without such role. S.E. denotes the bootstrap standard error (1,000 replications), yielding consistent results; Radius matching employed a 0.01 caliper, while kernel matching used the default bandwidth.

The size of the matched differences varied across dimensions. The largest difference was observed for engaging with others, followed by open-mindedness and collaboration, whereas the smallest difference was found for emotional regulation. Results from radius matching and kernel matching were consistent with the K-nearest neighbors estimates, indicating that the positive association between student leadership and social and emotional competence was robust across alternative matching specifications.

### 4.4 Mediation analysis

Bootstrap mediation analyses were conducted to examine whether teacher-student interaction, peer interaction, and learning engagement served as potential role-practice pathways linking student leadership to social and emotional competence. As illustrated in Figure 3, the mediation model included three parallel pathways from student leadership to social and emotional competence through teacher-student interaction, peer interaction, and learning engagement.

**Figure 3:**
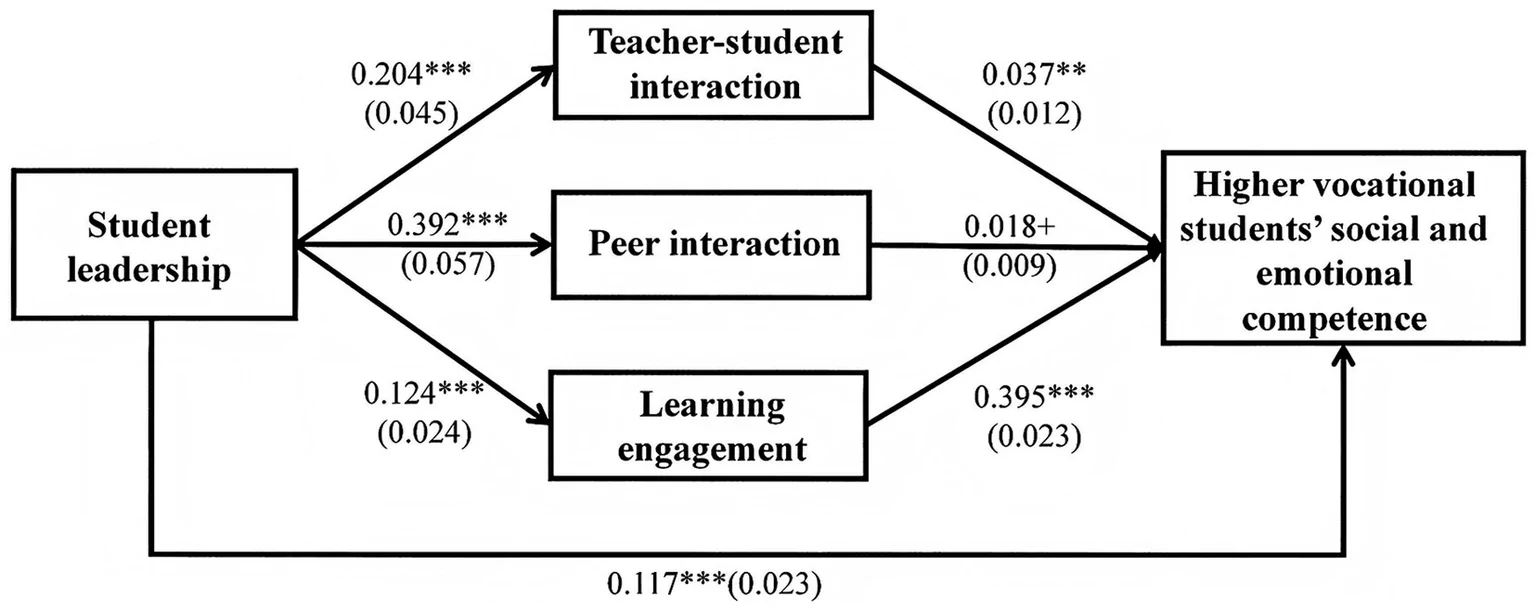
Potential pathways linking student leadership to higher vocational students’ social and emotional competence.

As shown in Table 4, all three indirect effects were statistically significant, as their 95% confidence intervals did not include zero. Teacher-student interaction partially mediated the association between student leadership and social and emotional competence, with an indirect effect of 0.021, 95% CI [0.010, 0.032], accounting for 11.73% of the total effect. Peer interaction also showed a significant indirect effect of 0.022, 95% CI [0.012, 0.032], accounting for 12.29% of the total effect. The indirect effect through learning engagement was larger, with an effect value of 0.052, 95% CI [0.031, 0.073], accounting for 29.05% of the total effect.

**Table 4:** Bootstrap test for the relative indirect effect of teacher-student interaction, peer interaction, and learning engagement.

Variable	Type of effect	Efficiency estimate	Boot SE	Boot LLCI	Boot ULCI	Proportion of total effect
Teacher-student interaction	Total effect	0.179***	0.027	0.133	0.226	100%
	Direct effect	0.158***	0.027	0.112	0.204	88.27%
	Indirect effect	0.021***	0.006	0.010	0.032	11.73%
Peer interaction	Total effect	0.179***	0.024	0.132	0.227	100%
	Direct effect	0.157***	0.025	0.109	0.205	87.71%
	Indirect effect	0.022***	0.005	0.012	0.032	12.29%
Learning engagement	Total effect	0.179***	0.024	0.132	0.226	100%
	Direct effect	0.127***	0.022	0.084	0.170	70.95%
	Indirect effect	0.052***	0.011	0.031	0.073	29.05%

Among the three pathways, learning engagement accounted for the largest share of the total effect, whereas teacher-student interaction accounted for the smallest share. This pattern suggests that learning engagement may be the most prominent motivational pathway linking student leadership to social and emotional competence, while teacher-student interaction and peer interaction also serve as significant relational pathways. H2a, H2b, and H2c were therefore supported. Given the cross-sectional design, these mediation results should be interpreted as potential explanatory pathways rather than confirmed causal mechanisms.

## 5 Discussion

### 5.1 Interpretation of the main findings

The present study examined whether student leadership is associated with higher vocational students’ social and emotional competence and further explored the relational and motivational pathways underlying this association. After reducing observable selection bias through propensity score matching, the results showed that students holding student leadership roles reported higher overall social and emotional competence than comparable students without such roles. Similar patterns were found across task performance, emotional regulation, collaboration, open-mindedness, and engaging with others. This result supports H1. It also differs from Zhou (2014), who noted that student leadership may generate psychological pressure and may even hinder the development of students’ social and emotional competence under certain conditions. The present findings are more consistent with research linking student leadership to communication, organization, responsibility-taking, academic development, and later career-related advantages (Deng et al., 2020; Cui et al., 2022; Qian, 2025; Rosch and Collins, 2017).

A useful perspective for interpreting the matched association between student leadership status and social and emotional competence is symbolic status. In Chinese higher vocational colleges, student leaders often occupy a visible position between institutional administration and student self-governance. This position may bring recognition, expectations, and responsibility from teachers and peers (Du, 2016; Ke and Li, 2020). From the perspective of symbolic capital, such recognition may become a psychological and social resource that supports students’ self-efficacy, responsibility, and role identity (Bourdieu, 1986).

A closer look at the dimensions suggests that student leadership may be more strongly related to socially situated aspects of social and emotional competence than to more internalized aspects. The largest matched difference appeared in engaging with others, while emotional regulation showed the smallest difference. This pattern is theoretically reasonable. Student leadership creates frequent opportunities for communication, coordination, and public participation, all of which are closely connected to engaging with others. By comparison, emotional regulation may be shaped by longer-term personality, family, and developmental factors and may therefore be less directly responsive to leadership-related experiences. The result echoes recent discussions that social and emotional competence is contextual, practical, and developmental rather than a fixed individual trait (Wang et al., 2024; Yang et al., 2025).

### 5.2 Role-practice pathways from a self-determination theory perspective

The mediation findings complement the status-based interpretation by highlighting relational and motivational processes associated with students’ leadership participation. At the institutional level, one pathway may operate through communication with counselors. In Chinese higher vocational colleges, counselors are closely involved in class management, student affairs, academic guidance, and broader student support, making them an important point of contact in students’ everyday college experience. Student leaders, in particular, often communicate with counselors about class affairs, academic tasks, student needs, and activity arrangements. These encounters are not only administrative exchanges. They also expose students to feedback, recognition, responsibility, and problem-solving situations that may help them interpret themselves as more capable participants in school life. This interpretation is consistent with Self-Determination Theory, which emphasizes the developmental importance of competence-supportive environments (Ryan and Deci, 2000; Ryan and Deci, 2020). In this sense, student leadership may make such competence-supportive experiences more frequent and role-specific. Rather, it suggests that student leadership may make such competence-supportive experiences more frequent and more role-specific. This finding also resonates with studies showing that high-quality teacher-student relationships and learning environments are closely related to students’ social competence and socio-emotional development (Magro et al., 2024; Wang et al., 2025).

At the peer level, participation in collective activities provides another setting in which these relational processes may unfold. Club activities bring students into recurring contact with classmates and create opportunities for communication, cooperation, shared participation, and the negotiation of different roles within a group. From a Self-Determination Theory perspective, these peer-based experiences are relatedness-oriented because they may strengthen students’ sense of connection and belonging (Ryan and Deci, 2020). This helps explain why student leadership is especially associated with collaboration and engaging with others.

Learning engagement appears to be the strongest pathway in the present study. Compared with teacher-student interaction and peer interaction, learning engagement accounted for the largest proportion of the indirect association between student leadership and social and emotional competence. This finding suggests that the developmental relevance of student leadership is not limited to interpersonal interaction. It may also operate through students’ active involvement in school life. Student leaders are often more visible within class communities and may be expected to serve as role models in both learning and collective affairs. Such visibility and responsibility may encourage greater persistence, participation, and self-regulated engagement. This interpretation is consistent with recent studies emphasizing that learning engagement is closely related to students’ academic adaptation, well-being, and broader developmental outcomes (Christenson et al., 2012; Collie and Martin, 2024). In the present study, learning engagement can therefore be understood as a motivational pathway through which student leadership is connected with social and emotional competence. At the same time, the three mediators together accounted for approximately 53% of the estimated total association, suggesting that the developmental relevance of student leadership is unlikely to be fully captured by the relational and motivational processes examined here. Other pathways may include leadership self-efficacy and identity development at the individual level, as well as the expansion of social networks at the relational level; these mechanisms were not measured in the present study and should be examined directly in future research.

Recent studies have increasingly emphasized the relational and motivational foundations of social and emotional competence. Wang et al. (2024) showed that support for students’ basic psychological needs is associated with better social and emotional competence across cultures, highlighting the relevance of Self-Determination Theory to the development of social and emotional competence. Wang et al. (2025) further demonstrated that university students’ social and emotional competence is closely related to the teaching and learning environment, especially active learning, good teaching, and clear goals. Similarly, Magro et al. (2024) found that teacher-student relationship quality is positively associated with students’ social competence with peers. These studies help explain why teacher-student interaction, peer interaction, and learning engagement matter in the present study. The present study, however, places these relational and motivational factors within the specific role-based context of student leadership rather than treating them only as general features of the learning environment.

### 5.3 Theoretical contributions

Compared with prior research, which has mainly linked student leadership to academic performance, organizational skills, responsibility, or later labor-market outcomes (Deng et al., 2020; Cui et al., 2022; Qian, 2025), the present study focuses on social and emotional competence as a multidimensional developmental outcome.

First, it reframes student leadership as a role-based developmental context. In much of the previous literature, student leadership has been treated mainly as a formal position or as an indicator of student status. This study interprets student leadership through the complementary perspectives of symbolic status and role practice. The distinction helps explain why the same leadership position may function as a source of recognition and expectations, while also generating repeated opportunities for communication, coordination, and responsibility-taking.

Second, this study extends the application of Self-Determination Theory to the context of student leadership. Existing SDT-based studies have often focused on teacher support, classroom climate, learning motivation, or need-supportive environments. The present study suggests that student leadership may also create relational and motivational experiences that are theoretically relevant to competence-supportive, relatedness-oriented, and autonomy-related developmental conditions. In this sense, students are not only passive recipients of educational support. They may also become active participants in school-based role practices that expose them to feedback, cooperation, and meaningful engagement.

Third, this study provides a more integrated explanation of how student leadership is linked to social and emotional competence. The findings suggest that the association is not produced by a single pathway. Rather, it may reflect the combined operation of teacher-student interaction, peer interaction, and learning engagement. This echoes recent research emphasizing the synergistic effects of multiple social and psychological resources in the development of social and emotional competence (Yang et al., 2025). For higher vocational education, this perspective is particularly important because students’ development is embedded in both institutional arrangements and everyday role participation.

### 5.4 Practical implications

The findings suggest several tentative implications for higher vocational colleges. First, student leadership should not be regarded only as an administrative arrangement for assisting teachers and managing class affairs. Its developmental value depends on whether leadership roles provide meaningful opportunities for communication, cooperation, responsibility-taking, and learning engagement. Given the observed associations with these relational and motivational processes, colleges may consider providing structured guidance for student leaders, for example by helping them reflect on what they learn from routine tasks and connect administrative duties with broader developmental experiences.

Second, the findings also raise the question of how leadership-related developmental opportunities might be made accessible to a broader range of students, rather than being concentrated among those who already possess stronger social resources or higher visibility. If leadership opportunities are concentrated among already advantaged students, they could potentially reinforce existing inequalities rather than broaden developmental opportunities. One possibility worth exploring is the use of rotating responsibilities, project-based teamwork, and short-term leadership tasks, which may allow more students to experience leadership-like role practice without necessarily holding formal titles.

Third, the mediation findings suggest that attention to the quality of interactions associated with student leadership may also be relevant. Teacher-student interaction, peer interaction, and learning engagement were all significant pathways in this study. Rather than implying that assigning leadership responsibilities alone will generate developmental benefits, these findings suggest that teachers and counselors may consider accompanying leadership roles with feedback, emotional support, task guidance, and fair recognition. They may also remain attentive to whether leadership responsibilities are accompanied by excessive burden or pressure, particularly among students with limited support.

### 5.5 Limitations and future research directions

Several limitations should be considered when interpreting the findings. First, although propensity score matching was used to reduce observable selection bias, the study was based on cross-sectional, single-source self-report data. Therefore, the results cannot establish a definitive causal relationship between student leadership and social and emotional competence. Students selected into leadership roles may already differ from non-leaders in prior motivation, personality, teacher relationships, social confidence, or family support. Although Harman’s single-factor test did not indicate a dominant single factor, common method bias cannot be completely ruled out. Future research should use longitudinal, multi-source, or stronger quasi-experimental designs to distinguish selection effects from role-socialization effects more clearly.

Second, student leadership was measured as a binary indicator of whether students held a formal leadership position. This measure enabled us to examine the association attached to formal leadership status, but it did not directly distinguish the symbolic meaning attached to that status from the specific role practices through which leadership is enacted. Accordingly, symbolic status and role practice are used in the present study as complementary explanatory perspectives for interpreting the matched association and the observed mediating processes, respectively, rather than as two separately measured mechanisms. Future research could develop multidimensional measures that assess symbolic-status characteristics and role-practice characteristics, such as task type, duration, intensity, and specific responsibilities. Such measures would allow the two theoretically distinguished processes to be examined more directly.

## 6 Conclusion

This study examined the association between student leadership and social and emotional competence among higher vocational students in Hubei Province, China. Using propensity score matching to reduce observable selection bias, the results showed that students with student leadership reported higher levels of social and emotional competence than matched peers without such roles. This pattern was observed across overall social and emotional competence and its five dimensions.

The mediation results further suggested that teacher-student interaction, peer interaction, and learning engagement served as potential role-practice pathways linking student leadership to social and emotional competence, with learning engagement accounting for the largest share of the indirect association. Taken together, the findings suggest that the developmental relevance associated with formal student leadership may lie both in the recognition attached to holding a leadership position and in the relational and motivational experiences accompanying leadership participation.

By focusing on higher vocational students, this study extends research on social and emotional competence to a vocational education context and offers a psychological explanation of how student leadership is linked to students’ relational, motivational, and developmental experiences in school life.

## Data Availability

The raw data supporting the conclusions of this article will be made available by the authors, without undue reservation.
